# NKG2D Ligands–Critical Targets for Cancer Immune Escape and Therapy

**DOI:** 10.3389/fimmu.2018.02040

**Published:** 2018-09-11

**Authors:** Dominik Schmiedel, Ofer Mandelboim

**Affiliations:** The Lautenberg Center for General and Tumor Immunology, The BioMedical Research Institute Israel Canada of the Faculty of Medicine, The Hebrew University Hadassah Medical School, Jerusalem, Israel

**Keywords:** NKG2D, NKG2D ligands (NKG2DL), cellular stress response, cancer therapy, immunotherapy, post-transcriptional regulation, post-translational regulation, shedding

## Abstract

DNA damage, oncogene activation and excessive proliferation, chromatin modulations or oxidative stress are all important hallmarks of cancer. Interestingly, all of these abnormalities also induce a cellular stress response. By upregulating “stress-induced ligands,” damaged or transformed cells can be recognized by immune cells and cleared. The human genome encodes eight functional “stress-induced ligands”: MICA, MICB, and ULBP1-6. All of them are recognized by a single receptor, NKG2D, which is expressed on natural killer (NK) cells, cytotoxic T cells and other T cell subsets. The NKG2D ligand/NKG2D-axis is well-recognized as an important mediator of anti-tumor activity; however, patient data about the role of NKG2D ligands in immune surveillance and escape appears conflicting. As these ligands are often actively transcribed, tumor cells are urged to manipulate the expression of these ligands on post-transcriptional or post-translational level. Although our knowledge on the regulation of NKG2D ligand expression remains fragmentary, research of the past years revealed multiple cellular mechanisms that are adopted by tumor cells to reduce the expression of “stress-induced ligands” and therefore escape immune recognition. Here, we review the post-transcriptional and post-translational mechanisms by which NKG2D ligands are modulated in cancer cells and their impact on patient prognosis.We discuss controversies and approaches to apply our understanding of the NKG2D ligand/NKG2D-axis for cancer therapy.

## The family of stress-induced ligands comprises high diversity on RNA and protein level

The NKG2D receptor is, in several aspects, an outstanding immune receptor of major interest in research and immunotherapy: First, the NKG2D receptor is expressed on lymphocytes both of the innate immune system, Natural Killer (NK) cells, as well as cytotoxic, CD4 or γδ T cells, which are assigned to the adaptive branch of the immune system (1, 2). Being considered a genuine activating receptor on NK cells, NKG2D acts as a co-stimulatory receptor on T cells (1, 3). Thereby, NKG2D receptor triggering induces not only cytotoxicity (4), but also drives cytokine production (5–7), or impacts T cell differentiation and expansion (8, 9). Second, the NKG2D receptor recognizes eight different ligands in humans, MICA, MICB and ULBP1-6 (10). Collectively, these are termed “stress-induced ligands” since they are differentially expressed after different cellular stresses. This redundancy facilitates the immune surveillance: NKG2D alone can recognize cell suffering from infection, DNA damage, fluctuating oxygen levels, excessive proliferation with active tumor-promoting signaling, or heat shock. NKG2D and its ligands are therefore key proteins to mount an immune response against unhealthy cells (10). However, it must be noted that expression occurs under certain conditions also on healthy cells, especially by immune cells for immunoregulatory purposes (11, 12). Third, the NKG2D ligand-NKG2D axis is widely recognized as anti-tumorigenic checkpoint. NKG2D-expressing immune cells are believed to reject transforming cells prior to immune-editing, which is a prerequisite for immune escape.

However, patient data of the past years revealed conflicting data and it appears that the importance of the NKG2D receptor in tumor immune surveillance and escape is far more complex as compared to other receptor-ligand interactions we are aware of (13).

Altogether, the wide range of NKG2D-expressing immune cells, the diverse ligand repertoire and its role in cancer therapy renders this receptor as an exceptional candidate for basic and applied cancer research.

## Differences and similarities in the NKG2D ligands in RNA and protein

The eight NKG2D ligands all belong to the family of MHC class I–like proteins. They share some degree of conservation, yet, they have distinct differences in their promoters, their RNA and protein sequences. Consequently, also their regulation is oftentimes unique and independent from each other.

As a variety of stresses induces differential expression of proteins of this family, it is not surprising that diverse cancer-associated transcription factors were previously shown to affect the expression of NKG2D ligands. Prominent examples of inducers of NKG2D ligand transcription are p53, that binds the promoter regions of ULBP1 and ULBP2 following DNA damage (14), Sp family transcription factors that influence the expression MICA, MICB and ULBP1 in proliferating cells (15, 16), or heat shock transcription factor 1 (HSF1) that binds its respective heat-shock elements in the promoters of MICA and MICB, respectively (17). An elaborate overview on the transcriptional regulation in humans and mice is given elsewhere (18). However, it is very clear that transcription is only the first controlled step in a multilayer of regulations that enables effective and fast induction of protein expression when required but suppresses undesired, excessive protein expression on healthy cells.

**Figure 1:**
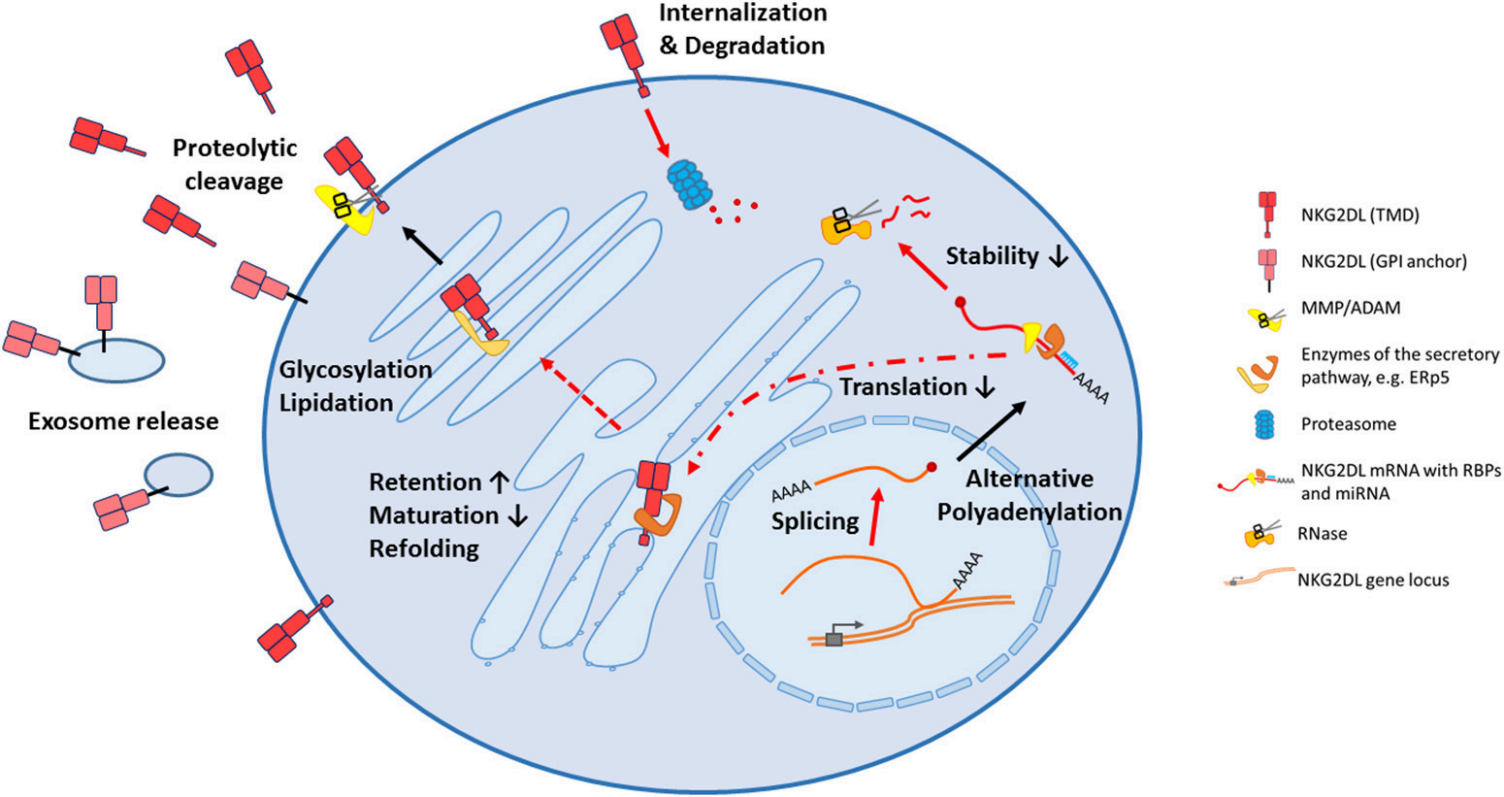
All stages of biogenesis of NKG2D ligands can be affected in cancer cells. Following transcription, mRNA processing can be altered affecting splicing and alternative adenylation, therefore different isoforms of a ligand can be produced. After export to the cytoplasm, mRNA translation can be inhibited by miRNAs, and decay is frequently induced by RNA binding proteins. During their trafficking in endoplasmatic reticulum and golgi apparatus, the NKG2D ligands can be refolded, by instance with the help of the thioisomerase ERp5, or differentially modified by glycosylations or lipidations. Potentially, some of these modifications contribute to intracellular retention by an impaired protein maturation. Certainly, these alterations change the biological properties of these ligands once they reach the surface, with the consequence that their release from the cell surface by shedding or release in exosomes is facilitated. Alternatively, the can also be internalized and degraded by the proteasome. NKG2DL, NKG2D ligand; TMD, transmembrane domain; GPI, glycosylphosphatidylinositol; MMP, matrix metalloprotease; ADAM, a disintegrin and metalloprotease; RBP, RNA binding protein; miRNA, microRNA.

**Figure 2:**
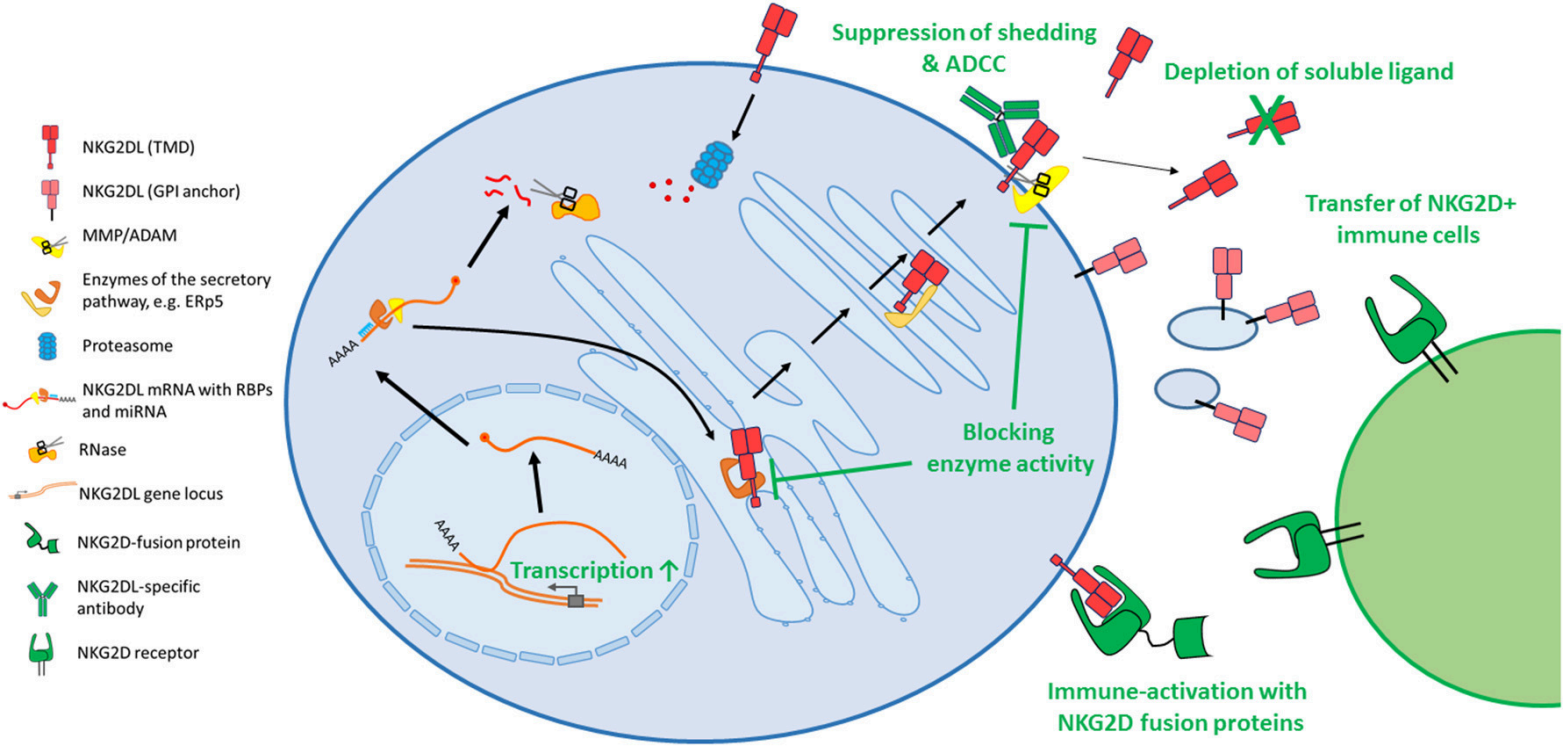
Diverse approaches attempt to target the NKG2D axis for cancer therapy. As most cytotoxic drugs induce or constitute a cellular stress, several classes of drugs induce expression of the stress-induced NKG2D ligands, amongst DNA damaging agents, proteasome inhibitors or histone deacetylase inhibitors. In order to decrease shedding of these ligands, small molecule inhibitors targeting matrix metalloproteases were developed. Other enzymes, which are involved in protein maturation, also pose potential drug targets. Antibodies can bind the surface MICA and prevent shedding and induce ADCC. Others bind and block soluble ligands and prevent their harmful binding to immune cells. Similarly, also apheresis can reduce the load of shed ligands in serum of cancer patients. To activate the immune system toward NKG2D ligand expressing tumor cells, diverse fusion proteins were created that contain the extracellular domain of NKG2D and are linked to IL-15, anti-CD3 or an Fc portion to induce ADCC, or others. Also, the transfer of NKG2D expressing immune cells, like bone marrow grafts, donor NK cells or genetically modified T cells are approaches to fight NKG2D ligand expressing tumors. NKG2DL, NKG2D ligand; TMD, transmembrane domain; GPI, glycosylphosphatidylinositol; MMP, matrix metalloprotease; ADAM, a disintegrin and metalloprotease.

Several mechanisms how these ligands are regulated on post-transcriptional level were disclosed in the past few years that will be discussed more thoroughly below. Unsurprisingly, these important immune-modulatory molecules are complexly and mostly independently regulated and research is just on the verge of deciphering underlying regulatory networks. In the first part of this review, we will summarize known cellular, post-transcriptional mechanisms that impact NKG2D ligand expression that may be hijacked by cancer cells to evade from NKG2D-mediated surveillance. An overview on the different mechanisms is provided in Figure 1. As the biology of ULBP4 (RAET1E), ULBP5 (RAET1G), and ULBP6 (RAET1L) and their role in tumor immunity is poorly understood, we will focus on the well-studied five ligands MICA, MICB, ULBP1-3. In the second part, we will provide an overview on current approaches to use these ligands for cancer therapy which are summarized in Figure 2.

## Regulatory circuits controlling NKG2D ligand expression

Due to the fact that post-transcriptional regulation mostly occurs within the untranslated regions (UTR) spanning the coding sequence upstream (5′UTR) or downstream (3′UTR) of the coding sequence, we analyzed similarities of the common variants and assessed sequence homologies using MUSCLE (19); however, we want to stress that annotations differ between databases. Also, sequences are still undergoing updates. Below, we refer to sequences found in the NCBI nuccore database (as of July 9th, 2018; NM_000247.2, NM_005931.4, NM_025218.3, NM_025217.3, NM_024518.2).

All 5′UTRs of these mRNA transcripts of all NKG2D are fairly short (below 100 nucleotides). However, the 3′ UTRs, which is considered the more important site on RNA regulation, shows striking differences: MICA and MICB share 90% homology, but about 1,000 nucleotides of the 3′UTR of MICB are missing in MICA. Similarly, ULBP1 and ULBP3 have extremely long UTRs of about 2,400 nucleotides, which are overall over 90% homologous. However, both UTRs contain unique regions stretching over 300 nucleotides that are not shared and therefore pose potential sites for differential regulation.

The ULBP2 3′ UTR consists of only about 550 nucleotides. It contains many single nucleotide exchanges when compared to ULBP1 and ULBP3, with an overall homology of 70%. Interestingly, only the beginning and the end of the sequence show conservation, whereas the sequences in between are largely missing.

Although single nucleotide exchanges exist, the observed differences suggest that the diversification in the RNA regulation following gene duplication is driven by deleterious events in the UTRs.

### Post-transcriptional regulators

MicroRNAs (miRNA or miR) were the first molecules described to impair the expression of the NKG2D ligands MICA, MICB and ULBP2 in cancer cells on mRNA level (20–24). In part, these miRNAs were shown to be overexpressed in the tumor itself (“oncomiRs”), like miR-93 that targets both MICA and MICB, but also in metastasis-associated miRNAs (“metastamiRs”), like miR-10b, that targets MICB expression (21). Interestingly, several binding sites for cellular miRNAs are overlapping with viral miRNAs (20), suggesting that the necessity to regulate stress-induced ligands using miRNA surpasses the need to evolve these sites to fight viral infections more efficiently. Next to the classical role of miRNAs to suppress protein translation, it was recently demonstrated that the 3′ UTR of the stress-induced ligand MICA can switch from a short to a long version by means of alternative polyadenylation in dependence of miRNA binding (24).

What is the role of miRNAs in the regulation of NKG2D ligands in healthy conditions? One theory is, that miRNAs are capable to inhibit the translation of NKG2D ligand protein, if only little mRNA is present, for instance due to mild stresses. A minor trigger is therefore insufficient to render a healthy cell as a target for immune cell attack. According to this line of thought, miRNAs work as a “buffering capacity” and a fine-tuner of ligand expression that suppress ligand expression up to a certain threshold of stress (20).

Next to miRNAs, also RNA binding proteins (RBPs) were shown to interact with NKG2D ligand transcripts: MICB expression is affected by at least twelve confirmed RBPs that bind its 3′UTR and impact all post-transcriptional aspects like processing, turnover rate, localization or translation rate (25). Most of which suppress MICB expression and are therefore described as negative regulators. Recently, also a negative regulator binding the short 5′UTR of MICB was described (26). Interestingly, all described RBPs were shown not to affect the close relative MICA (25, 26). Also ULBP1 biogenesis in cancer cells is critically affected by RBPs (27), as is ULBP2 mRNA stability (28). For ULBP1 is was additionally reported that RBPs affect biogenesis of different isoforms alternative splicing (27).

Figure 1 gives a summary on the post-transcriptional regulations mentioned above. Still, pathways and critical players that affect the fate of the RNA transcripts of NKG2D ligands are only fragmentarily understood. Additionally, in many occasions, we don't know if discovered mechanisms are tumor-specific, or at least enhanced in cancer cells, or if they are simply part of the “healthy” RNA processing pathway for these ligands.

### Post-translational effects

Next to differences in the UTR regions of the ligands, major differences are also observable in the protein sequence. ULBP family members possess an α1/α2 domain structure, while MIC proteins possess an α1/α2/α3 structure (29). Although being classified as a MHC class I–like protein, both families lack peptide binding ability and association with β2-microglobulin (18, 29, 30). On top of this layer of diversity, over 100 MIC alleles and 16 ULBP allelic variants were discovered. Notably, MICA and MICB do not possess hypervariable regions like classical MHC molecules. Genetic shuffling and point mutations occur over all three domains (31, 32). Another notable distinctive feature is the membrane anchorage. ULBP1, ULBP3, ULBP6 and one prevalent allele of MICA, MICA^*^008, are attached to the membrane by a GPI (glycosylphosphatidylinositol) anchor (33), whereas ULBP4 and ULBP5, MICB alleles and all other allelic variants of MICA are embedded into the lipid bilayer with a transmembrane domain. Uniquely, ULBP2 can be expressed both as GPI- or transmembrane anchored protein (34). Whereas, the GPI-anchored ligands localize to lipid rafts, members possessing transmembrane domain appear not to do so (35). Altogether, these proteins belong to one of the most plastic families encoded in the human genome (33).

Yet, it is not understood to what extent differences in surface localization or affinities to NKG2D give rise to differential functional outcomes when triggering the receptor. Also, we don't understand yet why the MIC proteins, and MICA in in particular, are so superior regarding their potential to create new allelic variants compared to ULBP family members.

### Tackling the ligands on the protein level

Like outlined above, the regulation of the eight different ligands on RNA level is multilayered and largely non-redundant. Therefore, many cancer cells can't efficiently inhibit the biogenesis of the transcripts or suppress their translation.

However, options to prevent a translated ligand from being surface-expressed exist as well. These findings are summarized in Figure 1.

First, there are several reports showing that NKG2D ligands are indeed expressed but retained intracellularly (36, 37). However, until now, we lack understanding which proteins are involved in this retention process that is exploited by cancer but most likely also a cellular mechanism in ligand homeostasis (37). A recent report showed that hypoxic conditions modulate MICA glycosylation and thereby prevent surface expression (38). Accordingly, glycosylation and therefore protein maturation may be one contributor in this process.

Second, surface expressed ULBP1 can be internalized and degraded by the proteasomal pathway (39). Thereby, both the levels of surface expression but also the duration of the stress-response can be controlled.

Third, these ligands can be released from the tumor cell surface, a process termed “shedding.” Cells can shed ligands either by proteolytic cleavage or by releasing ligands in exosomes.

## Shedding-an efficient way to evade from NKG2D-mediated surveillance

Shedding constitutes a very beneficial mean for cancer cells to avoid surface expression of these ligands. Metalloproteases, most prominently ADAM10, ADAM17 (40, 41), and MMP14 (42) are frequently expressed in the tumor microenvironment but also on platelets (43), cleave and thereby remove MICA, MICB, or ULBP proteins from the tumor cell surface (44–48). The process of shedding is influenced by proteins that inhibit metalloprotease activity like TIMP3 (49), or that enable or facilitate the proteolytic cleavage, like the disulfide-isomerase ERp5 (50). Accordingly, high ERp5 and ADAM10 expression were shown to yield a high load of soluble NKG2D ligands in supernatants of primary cancer cell cultures (51). Ligands, which are linked to the membrane via a GPI-anchor, like ULBP1, ULBP3 or the MICA allele ^*^008, are frequently released in exosomes (52, 53). For MICA, palmitoylation was shown to be crucial for co-localization with the exosome-forming protein caveolin-1 and therefore for the incorporation in exosomes (54). Although both soluble and exosomal-released NKG2D ligands bind the NKG2D receptor and mediate receptor internalization, a stronger internalization of the receptor is induced by exosomal-released ligands, probably due to their ability to crosslink the receptor on the surface (55, 56).

Shedding provides several major advantages for the cancer cell in terms of immune evasion. First of all, if one or several of these ligands are released systemically into the bloodstream of patients, they are not cell surface-exposed and therefore unable to activate NKG2D receptor-bearing cells.

More importantly, the released ligands are still capable of binding the NKG2D receptor on NK or T cells. In consequence, the NKG2D receptor is internalized in both NK and CD8 T cells (57–59). NKG2D receptor internalization is a major downside of the promiscuity of the NKG2D receptor. Whereas, a diverse array of stresses can be recognized by a functional receptor, the shedding of a single ligand is sufficient to render immune cells blind to the entire ligand family. On top, chronic engagement of the NKG2D receptor was shown to downmodulate also the activity of other NK cell receptors (60, 61) which may be in part connected to the degradation of the CD3ζ signaling molecule that also impairs T cell activity (62). Therefore, shedding is a very powerful way to overcome NKG2D-mediated immune surveillance.

### NKG2D ligands as prognostic marker in cancer

Ultimately, these differences on RNA and protein level determine NKG2D ligand regulation and expression patterns and impact thereby their importance in tumor biology.

Histological analyses of tumor samples puzzled researchers and doctors alike for several years, as the expression of NKG2D ligands was sometimes favorable and sometimes unfavorable for disease prognosis–different studies appeared contradictory (63, 64). However, this diversity was due to the inability to discriminate soluble and membrane expressed ligands in histology. Today we know, that solely membrane-bound ligands on cancer cells are a positive predictor for patient survival (65). However, the levels of soluble stress-induced ligands in the serum cancer patients pose a valuable prognostic factor. First, they anti-correlate with NK and T cell activity; second, they correlate to staging of the disease and have an overall negative impact on patient survival (48, 65). In line with the decrease in immune cell activity due to soluble NKG2D ligands, checkpoint inhibition therapy using PD-1 antibodies in melanoma was found to be most effective in absence of shed ligands (66), supporting the view that NKG2D ligands need to be taken in consideration for therapies that are not “intentionally” involve the NKG2D-axis.

### NKG2D ligands-a promising target for immunotherapy

As surface-expressed NKG2D ligands promote tumor rejection and give a favorable survival prognosis, these ligands pose a promising therapeutic target for (immuno-) therapy. Diverse attempts to manipulate the expression of these ligands were undertaken in the past few years in order to harness the immune system against cancer. An overview of different strategies is given in Figure 2.

As most anti-cancer drugs act by inducing immediate cellular stress (with the ultimate goal to induce cell death), surface expression NKG2D ligands is frequently increased following treatment. Diverse compounds were identified that substantially increase stress-ligand expression and thereby render tumor cells more susceptible to immune cell attack. One prominent example is the histone deacetylase (HDAC) inhibitor valproic acid (67–69) which was shown to upregulate ligands *in vitro* and *in vivo* (68). But also other drugs like hydroxyurea (70), bortezomib (71), all-trans-retinoid acid (68) or sodium butyrate (72, 73) appear to enhance stress-ligand expression. Therefore, patients can benefit more of some regimens if the drugs not solely damage cancer cells but also lead to the loss of immune tolerance toward the tumor (73, 74). However, whereas valproic acid appears to increase only the level of membrane-bound but not of soluble ligand in cell cultures (69, 75), other HDAC inhibitors apparently induce metalloprotease expression and might therefore also increase shedding (76). Also, an impairment of NKG2D receptor expression in NK cells upon HDAC treatment was reported (77, 78). Future research in this field should address this issue and assess effects on NKG2D ligands more systematically and *in vivo*, as frequently used *in vitro* models don't reflect the complexity in the interplay of tumor cells, tumor microenvironment and NKG2D expressing immune cells which are also impacted by the treatment. It is important to choose proper models that may actually predict if NKG2D mediated immune surveillance can be restored, and if patients may actually benefit of these approaches.

Whereas induction of ligands might be beneficial to activate the immune system, many late-stage cancers release ligands in soluble form and are therefore inappropriate candidates for these kind of anti-tumor strategies. The use of different inhibitors of shedding proved the concept that soluble NKG2D ligands can be effectively reduced, and that their immune-disarming properties can be reversed. Examples are inhibition of the thioreductase ERp5 (50), or prevention of proteolytic cleavage by sheddases like MMP9 (79) or ADAM10 (80). Attempts of the past years yielded selective small molecule inhibitors for MMPs like ADAM10. These are considered for cancer-therapy also for other immune-modulatory purposes besides the manipulation of NKG2D ligands which are summarized elsewhere (81). In contrast, for other potential targets, like ERp5, solely unspecific inhibitors exist at present which therefore pose no therapeutic option in the near future.

But not every new target requires a new drug development: some clinically applied drugs appear to reduce shedding of ligands as a pleasant “side effect,” as shown for hypomethylating agents (49) or tyrosine kinase inhibitors (82).

However, we should bear in mind that sheddase activity is also important for the mounting immune response, for instance, the release of TNFα and fractalkine is mediated by ADAM17 (83, 84).

Recently, a new antibody was developed to prevent the shedding of both MICA and MICB in order to restore NKG2D receptor activity and induce better killing of tumor cells by inducing antibody dependent cellular cytotoxicity (ADCC) (85).

However, while inhibiting shedding will be helpful in many cancer patients, exosome release of ligands is another issue that will need to be addressed. Another promising attempt uses adsorption apheresis or antibodies to reduce soluble MICA levels in the serum to restore functionality of NKG2D-bearing immune cells. In consequence, NK cell activity was successfully restored following depletion of soluble NKG2D ligands in plasma of cancer patients (86, 87).

A third, commonly exerted approach to target NKG2D ligand expressing tumor cells is the development of fusion proteins that show promising anti-tumor effects in mouse models. By using the NKG2D extracellular domain (ECD) fused to an immune-activating component, diverse immune effector mechanisms can be targeted against NKG2D ligand expressing cells. By instance, a NKG2D receptor domain fused to the constant domain of an antibody mediates ADCC (88, 89) via engagement of Fc receptors on immune cells. The fusion of the ECD to a single chain targeting CD3 directs T cell immune responses against the tumor cells (90, 91). The fusion with cytokines like IL-21 or IL-15 can activate T and NK cell immunity in the tumor proximity and help tumor clearance (92–94).

Last but not least, hematopoietic stem cell transplantation (HSCT) and infusions of immune cells like NK cells or genetically modified T cells, were also studied with particular emphasis to the function of the NKG2D receptor. HSCT is frequently exerted to treat hematopoietic malignancies (95, 96). Due to conditioning regimens leading to cellular stress, transplanted NKG2D expressing cells are critically involved in graft vs. leukemia but also graft vs. host disease (97). Infusions of activated NK cells to treat cancer are generally considered safe (98), and can scavenge soluble MICA in the serum of cancer patients, thereby restoring NKG2D-mediated immune surveillance (99). Also, for several years, the anti-tumor efficacy of T cells with possessing a transduced NKG2D chimeric antigen receptor (CAR) with different signaling domains were assessed (100). However, whereas the efficacy of these engineered cells appears to be striking in mouse models (101–103), unwanted activation and fratricide (104) of CAR T cells combined with excess cytokine release (105, 106) pose severe problems that likely prohibit studies in patients. The sensitivity of NKG2D CAR T cells is apparently too high so also healthy cells pose targets and lead to excess CAR T cell activation.

## Conclusions

The past few years revealed a lot of new insights into the regulation of post-transcriptional and post-translational level of stress-induced ligands harboring an unforeseen complexity of regulation. We also gathered a wider understanding on how NKG2D ligands control immune responses by affecting immune cell activity, in health and disease, and opened paths how to use the NKG2D ligand axis for cancer therapy.

Yet, our understanding remains too fragmentary. Several regulatory mechanisms and factors can be attributed to determine the fate of a single ligand, but its importance for other members of the family is unknown. Certainly, our knowledge of factors affecting ligand biogenesis only scratches the surface of different layers of regulation. For instance, it appears that NKG2D ligands can be retained intracellularly, but we lack understanding how this takes place. Altogether, we miss a holistic picture, a systematic landscape of regulation, that determines which pathways (instead of single proteins or RNAs) regulating NKG2D ligands deteriorate in auto-inflammation or cancer. Such a landscape would also provide new targets, disclose new therapeutic options to harness the NKG2D axis in cancer therapy. On top, we still don't understand well, if and how different NKG2D ligands modulate immune responses differently. Can we utilize NKG2D ligands not only to kill tumor cells directly but also to orchestrate the immune response by impacting the crosstalk of immune cells? A very recent report disclosed that the NKG2D receptor triggered by ULBP2 exhibits a different nanoscale organization on the surface compared to an engagement with MICA, also leading to different functional outcomes (107), thereby giving insights to previously reported differential effects on NKG2D receptor endocytosis by binding to different ligands (108). Future studies will need to address these differences to understand the role of eight different ligands in immune homeostasis under healthy conditions as well as in cancer and autoimmunity. However, we should also bear in mind that the evolution of this diversity might be triggered by pathogens, and viruses in particular, that modulate stress-induced ligands as a mean of immune evasion (109).

Nonetheless, what we know now gives hope that the NKG2D axis might be a game changer—at least for some cancer patients. Arising methods to inhibit or deplete of soluble ligands may become more effective and easily applicable, neutralizing a strong immune inhibitor. Patients, that currently fail to mount an immune reaction when receiving checkpoint inhibitors, may regain responsiveness. Combinatorial approaches with NKG2D ligands are a very promising target to overcome the immune-suppressive tumor environment and re-activate the immune system for an anti-tumor reaction.

The next few years will show, how far we can reach out utilizing the NKG2D receptor in therapy, but we should not lose focus to advance also most basic knowledge on the NKG2D ligand and receptor axis, as every new evidence will help us to personalize (NKG2D mediated) therapies.

## Author contributions

DS outlined, wrote, referenced the manuscript, and prepared the figures. OM supervised and carefully edited the work.

### Conflict of interest statement

The authors declare that the research was conducted in the absence of any commercial or financial relationships that could be construed as a potential conflict of interest.
